# Office-based Exercise Therapy as a Non-pharmacological Treatment for Discogenic Low Back Pain among Army Staff

**Published:** 2018-12

**Authors:** Reza ALIZADEH, Ardalan SHARIAT, Noureddin NAKHOSTIN ANSARI, Ramin KORDI, Joshua A. CLELAND, Azadeh HAKAKZADEH, Mehdi KARGARFARD

**Affiliations:** 1. Dept. of Anesthesiology and Pain, AJA University of Medical Sciences, Tehran, Iran; 2. Sports Medicine Research Center, Neuroscience Institute, Tehran University of Medical Sciences, Tehran, Iran; 3. Dept. of Physiotherapy, School of Rehabilitation, Tehran University of Medical Sciences, Tehran, Iran; 4. Neuromusculoskeletal Research Center, Iran University of Medical Sciences, Tehran, Iran; 5. Dept. of Physical Therapy, Franklin Pierce University, Manchester, New Hampshire, USA; 6. Dept. of Exercise Physiology, Faculty of Sport Sciences, University of Isfahan, Isfahan, Iran

## Dear Editor-in-Chief

Musculoskeletal issues are extraordinarily overwhelming in industrialized nations and reportedly influence between 70%–80% of adults at some point during their lives (1). Over course of one’s life, Low Back Pain (LBP) is exceptionally common (2). The Worldwide Burden of Illness reports LBP to be one of the top 5 conditions contributing to disability (3). Persistent LBP is complicated since not all deteriorated or herniated disks result in symptoms. Disk degeneration is a normal process starting around the third decade of life. In some circumstances, the disk can be a symptom generator (4). One of the greatest impacts of LBP is a loss of work productivity (5). Delay in return to work results in considerable costs. These costs have been reported exceed $50 billion per year in the US, $11 billion in the UK, and $5 billion in the Netherlands (6). In most individuals with LBP, there is no evident spinal pathology.

Approximately 40% of Discogenic LBP (DLBP) might be associated with degeneration of the nucleus pulposus, tearing of the annulus, and intradiscal changes (7). The treatment of DLBP remains disputable. Though prevalent, there are no specific medicines for DLBP (8). Many physicians are no longer prescribing medications and have resorted to physical treatment.

Considering the busy schedule of army staff, and side effects of surgery, it seems vital to identify a cost-effective package of exercise training for pain relief and treatment of army staff with DLBP. For this purpose, there will be two important questions: what is the prevalence of DLBP among army staff in Asian countries as most of the previous researches were conducted in Western and American countries. Secondly, what is the optimal package of exercise training for treatment and pain relief among this population?

We strongly suggest researchers continue to examine the prevalence of DLBP among army staff. Importantly, it may be necessary to develop a specific package of office/home-based exercise therapy for this population.

A package of office-based exercise training with therapeutic aims was recently suggested, in the treatment of neck, shoulder and lower back pain among office workers (9). This package was suggested to be performed 3 times a week, during working hours. This package was recognized and introduced as a feasible and cost-effective therapeutic pattern of exercise training for office workers (10) for treatment and pain relief without side effects. This package is based on guidelines from the American College of Sports Medicine Williams’s flexion exercises, and Mackenzie extension exercises. The pain related to lower back could be also associated with psychological issues; the package may be needed to include psychological training. However, it is still not clear, if this type of package could be effective for army staff with DLBP. Clinicians should work in the future to determine whether the office-based exercise package for office workers is effective in army staff with DLBP.
